# Recent Highlights of Metabolomics in Chinese Medicine Syndrome Research

**DOI:** 10.1155/2013/402159

**Published:** 2013-11-04

**Authors:** Ai-hua Zhang, Hui Sun, Shi Qiu, Xi-jun Wang

**Affiliations:** National TCM Key Laboratory of Serum Pharmacochemistry, Key Laboratory of Metabolomics and Chinmedomics, Department of Pharmaceutical Analysis, Heilongjiang University of Chinese Medicine, Heping Road 24, Harbin 150040, China

## Abstract

Chinese medicine syndrome (CMS, “ZHENG” in Chinese) is an understanding of the regularity of disease occurrence and development as well as a certain stage of a comprehensive response of patients with body condition. However, because of the complexity of CMS and the limitation of present investigation method, the research for deciphering the scientific basis and systematic features of CMS is difficult to go further. Metabolomics enables mapping of early biochemical changes in disease and hence provides an opportunity to develop predictive biomarkers. Moreover, its method and design resemble those of traditional Chinese medicine (TCM) which focuses on human disease via the integrity of close relationship between body and syndromes. In the systemic context, metabolomics has a convergence with TCM syndrome; therefore it could provide useful tools for exploring essence of CMS disease, facilitating personalized TCM, and will help to in-depth understand CMS. The integration of the metabolomics and CMS aspects will give promise to bridge the gap between Chinese and Western medicine and help catch the traditional features of CMS. In this paper, particular attention will be paid to the past successes in applications of robust metabolomic approaches to contribute to low-molecular-weight metabolites (biomarkers) discovery in CMS research and development.

## 1. Introduction 

Metabolomics is a powerful new technology that allows for the assessment of global metabolic profiles in easily accessible biofluids and biomarker discovery in order to distinguish between diseased and nondiseased status information [1]. Combining metabolomic data with multivariate data analysis tools allows us to study alterations in metabolic pathways following different perturbations. Metabolite changes that are observed in diseased individuals as a primary indicator have been an important part of clinical practice. Most clinical chemistry tests available today rely on old technologies, and these tests are neither sensitive nor specific for any particular disease and traditional markers only increase significantly after substantial disease injury [2]. Therefore, more sensitive markers of disease are eagerly needed, particularly, for the early detection of disease, and highly sensitive and specific biomarkers as primary indicators are relatively more useful [3–5]. Discovery of clinically relevant biomarkers for diseases has revealed metabolomics has potential advantages that classical diagnostic approaches do not.

Traditional Chinese medicine (TCM) has been practiced for thousands of years and attracted worldwide interest, based on a holistic view that cure diseases through establishment of equilibrium in the human life [7]. The major advantage of TCM is treatment based on the treatment of differential syndrome (TDS, diagnosis, and treatment based on an over-all symptoms and signs) [8]. Syndrome in TCM is the comprehensive analysis of clinical information gained by the four main diagnostic TCM procedures: observation, listening, questioning, and pulse analysis [9]. Currently lack of an easy-to-use and inexpensive sampling method and lack of an accurate and portable platform to facilitate early syndrome detection are the major limitations that have prevented people from recognizing the full potential of TCM and have seriously hampered the development of clinical diagnostics. Fortunately, the integrative approach of metabolomics is in line with the holistic concept and practices of TCM and will gain a revolution in understanding of essence of CMS [10]. Some characteristic examples are presented to highlight the application of this platform to CMS research and development as well as some of the necessary milestones for moving TCM into mainstream health care.

## 2. Advantages of Metabolomics 

Metabolomics has recently moved into one of the cornerstones of postgenomics for the quantitative analysis and unbiased identification of small molecule metabolites within an organism, processes important in clinical medicine. It can incorporate the most advanced technology to provide the ideal platform for the discovery of biomarkers regarding multifactorial diseases. The metabolites could yield important information about a person's health or disease. More specifically, metabolomics has a global and noninvasive analysis of biomarkers that are indicators of normal biological or pathogenic process, or response to therapeutic intervention, thereby helping to monitor treatment response [11]. Intimately, these large-scale analyses of metabolites are bound to novel mass spectrometry (MS) technology analyzers with the combination of hyphenated techniques [12]. Approaches of either HPLC or UPLC online with MS have recently been employed and became increasingly popular [13]. The most commonly used noninvasive techniques are an essential requirement; thus urine is particularly and ideally suited for large-scale research of metabolomic analysis even in small volume. Metabolites that can serve as diagnostic markers have also increased, as has the fact that these biomarkers may be useful in following and predicting disease progression or response to therapy, some of which may be molecular targets for therapeutic intervention [14]. Over the last few years, the progression made in metabolomics has provided insightful information on disease development and disease onset prediction [15].

## 3. Bringing Metabolomics into the Forefront of TCM

TCM is one of the rarely existing ancient traditional medicines that hold systematic theories as well as preventative and therapeutic methods for diseases in practice. Nowadays, there is strong evidence that the TCM has increasing popularity throughout the world, which especially shows great superiority in early intervention, combination therapies, disease control, personalized medicine, and so forth [16]. The focuses of TCM science are on the patient rather than disease. The syndrome is one of the most important concepts and ingredients in the theoretical and clinical research of TCM. TDS is basic principle of understanding and treatment of diseases in TCM. Syndrome, key term in TCM theory, is an outcome after a careful analysis of all symptoms and signs. Syndrome embodies the integrity status of the whole body condition in a specific time, space, environment, and pathogenic factors. These factors include the genetic, physiological, pathological, psychological, spiritual, social, and other aspects of the individuals. Disease treatment based on the syndrome differentiation is to determine the appropriate treatment. TCM treatment of disease does not primarily focus on the “the similarities and differences of disease” but rather focus on the difference of syndrome through TDS and better understanding of disease. The introduction of powerful new technologies should greatly accelerate the pace of personalized TCM. In this context, there are many new opportunities and challenges for the TCM research in postgenome era. 

A syndrome is often understood in terms of the overall state of the body under the internal and external factors of the body combined with the environment, as well as changes with the course of development [17]. It is involved in multifactor and multilevel manners with the overall emergence of complex systems. Syndrome differentiation is the method of recognizing and diagnosing diseases or body imbalances by analyzing patient information based on TCM theories and experiences of doctors. The occurrence and development of a syndrome, which reflect pathological changes in some stages of disease development, inevitably affect human metabolism and change chemical substances in body fluids. From the perspective of systems biology, a syndrome may be a specific state in which protein networks and gene regulatory networks are “disturbed.” Such disturbances may be reflected by changes in the endogenous composition, which can be secreted into the blood and urine [18]. Metabolomics incorporates a “top-down” strategy to reflect the terminal symptoms of a whole system caused by interventions in the holistic context, assesses holistic metabolic profiles in easily accessible biofluids, and facilitates biomarker discovery. The distinct features of syndrome and diagnostic model have constantly challenged the methodology. Many problems still exist in current studies, such as the standard of CM syndrome differentiation, the design methodology, and criteria to assess the efficacy of interventions. Metabolomics has been gradually introduced to CMS studies, making it more scientific.

Combining the metabolomics with in-depth investigations of CMS mechanisms will enable a revolution in our understanding of disease pathology and will advance TCM. Metabolomics studies living systems from whole systems instead of isolated parts and opens up a novel opportunity to reinvestigate TCM. Adoption of metabolomics approach would do much help for exploring the scientific connotation of CMS and revolutionizing personalized TCM. There are new signs that the pursuit of both TCM and metabolomics will be a priority for people and advances paving the way towards personalized health care.

## 4. Metabolomic Dissection of CMS

The key problem in the CMS research is how to establish a new approach to fit the potential law of CMS. Fortunately, the rapid development of metabolomics technology platforms provides a methodological basis for deep understanding of the essence of CMS. Metabolomics takes a global or system thinking of the human body and appears to be the holistic view of TCM. Presently, a growing body of evidence demonstrates that metabolomics has been used to explore the particular metabolites and potential biomarkers and pathways of CMS, indicating that it is important to impact our understanding of the theory that is based on the evidenced TCM [19]. Particularly, for the early detection of disease, highly sensitive and specific biomarkers as primary indicators in biofluids are relatively more useful because those can be used for nonbiopsy tests. Analyzing and verifying the specifically early biomarkers of a disease, metabolomics enables us to better understand pathological processes and substance metabolic pathways. Compared with traditional diagnostic methods, even little changes of metabolites can help to detect early pathologic changes more sensitively. 

With the increasing pace of modern life, many people have been suffering from liver diseases commonly complicated by jaundice syndrome (JS) that is a common disease in China. JS, a common and fatal disease requiring early diagnosis and treatment, mainly involves two subtypes of TCM syndromes, YangHuang (YAH) and YinHuang (YIH). YAH is related to the dysfunction of liver, spleen, stomach, and gall bladder and seems to be acute viral hepatitis. YIH is the body function decline caused by excessive metabolic activity, like the case of obstructive jaundice. Measuring levels of alanine aminotransferase, aspartate aminotransferase, and so forth in blood samples have been the main diagnostic standard for JS. However, sensitivity of these markers is relatively low. Fortunately, metabolomics has been used to explore the particular metabolites, potentially diagnostic and prognostic biomarkers and pathways of syndrome. In an article currently published in* Mol Cell Proteomics *[2012; 11(8):370-80], Wang and coworkers used UPLC-Q-TOF-HDMS combined with pattern recognition methods to investigate a comprehensive metabolome of JS in order to establish specific metabolites phenotype and generate a better understanding of the pathophysiology [20]. Results indicate that JS related metabolites play an essential role in glutamate metabolism, synthesis, and degradation of ketone bodies, alanine, and aspartate metabolism, which are tightly correlated with the genes of neurotransmitters, hormones, and cytokines in the metabolites interaction network. Interestingly, 44 distinct metabolites identified from these pathways are in various stages of progress at the JS. Furthermore, vitamin B6 metabolism, tryptophan metabolism, arginine, and proline metabolism were also the top functions listed by MetaboAnalyst for YAH patient. Significant changes associated with YAH disease, defined as metabolite changes in YAH versus controls, were identified for 40 metabolites that are potential candidates for biomarkers. Additionally, steroid hormone biosynthesis, primary bile acid biosynthesis, cysteine, and methionine metabolism were all related to YIH. It is noteworthy that 49 metabolites together are important for the host response to YIH through target metabolism pathways. The results not only indicated that metabolomics had sufficient sensitivity and specificity to distinguish JS from healthy controls but also contributed to a further understanding of disease mechanisms.

Understanding syndromes is a core research to develop more efficient therapeutic strategies and diagnostic criteria for patients. Clinical evidence has shown that patients with liver-stagnation and spleen-deficiency syndrome (LSS) that characterized by metabolic disorder of body fluid, and causing liver failure and weakness. It is difficult to get outcome immediately and not particularly effective in separating cases of LSS from other non-LSS disorders. Development of biomarkers with higher sensitivity and specificity is waiting to emerge. Recent advances in metabolomic technology made it possible to identify the metabolites in the clinical samples and thus extensive efforts are now attempted to search for the biomarkers. Metabolite profiling of urine samples collected from patients with LSS and healthy controls was performed by UPLC-Q-TOF-HDMS in conjunction with multivariate data analysis and ingenuity pathway analysis that were used to select the metabolites to be used for the noninvasive diagnosis of LSS [21]. Twelve urinary differential metabolites contributing to the complete separation of LSS patients from matched healthy controls were identified involving several key metabolic pathways. Of note, of the differential metabolites, 4 metabolite markers could provide the effective diagnosis for human LSS. A prediction model was developed to indicate LSS, and an accuracy of 93.0% was obtained and suggested that metabolomics is highly effective in aiding biomarker identification and thus implies a new strategy in the diagnosis of LSS. These observations support that metabolomics is an ideal approach to reveal the scientific and intrinsic connotation of TCM syndromes also opens new perspectives to resolve special TCM issues.

## 5. Conclusion and Future Perspectives

Metabolomics is the comprehensive assessment of endogenous metabolites and attempts to systematically identify and quantify metabolites from a biological sample. Small-molecule metabolites have an important role in biological systems and represent attractive candidates to understand disease phenotypes. One area of considerable interest in the field of metabolomics is to detect potential biomarkers associated with diseases, and the metabolic profiling could provide global changes of endogenous metabolites of patients. The future goals for metabolomics are the validation of existing biomarkers, in terms of mechanism and translation to man, together with a focus on characterizing the individual healthcare. Under the guidance of the TCM theory, the metabonomics approach can be used to evaluate clinical syndromes and identify potential biomarkers [22]. The integral metabolomics is the best to fit the holistic concept of multitargets and systems of TCM theory; therefore it may be one of the best methods to study the TCM science. Widespread use of this technique would significantly advance the field of TCM by bridging the gap between Chinese and Western medicine. It is conceivable that the application of metabolomic platforms will eventually lead to the reconciliation and integration of TCM with contemporary medicine and systems medicine. In summary, integration of metabolomics-based diagnostic principles into the TCM would make it possible to interpret and explore the essence of personalized TCM and might be the direction to enable a revolution for future health care, and also perhaps it is time to embrace the arrival of “TCM-OMICS” era in Chinese medicine research.
